# The Discontinuation of Antibiotics in Patients with Chronic Obstructive Pulmonary Disease Exacerbation and a Positive Respiratory Viral Assay: A Single-center Retrospective Analysis

**DOI:** 10.7759/cureus.6491

**Published:** 2019-12-28

**Authors:** Nasheena Jiwa, Uzochukwu Ibe, Rohit Beri, Richard Feinn

**Affiliations:** 1 Pulmonary and Critical Care Medicine, University of Connecticut School of Medicine, Farmington, USA; 2 Cardiology, Danbury Hospital, Danbury, USA; 3 Pulmonary and Critical Care Medicine, St. Mary's Hospital, Waterbury, USA; 4 Statistics, Frank H. Netter MD School of Medicine, North Haven, USA

**Keywords:** respiratory infections, acute exacerbation of copd, viral infections, antibiotic stewardship, respiratory viral panel

## Abstract

Background

The use of antibiotics in chronic obstructive pulmonary disorder (COPD) exacerbations attributed to viral infections is observed in this study. The aim of this analysis is to describe the rate of discontinuation of antibiotics in patients who have an acute exacerbation of COPD (AECOPD) caused by viral infections, in turn encouraging the use of the respiratory viral panel in an effort to improve antibiotic stewardship at our facility.

Methods

A retrospective chart review was performed. A total of 92 patients were analyzed who had a positive respiratory viral polymerase chain reaction (PCR) (RVP) admitted for COPD exacerbations, of which 20 patients had a bacterial co-infection by a sputum analysis. Patients with a positive infiltrate on chest X-ray (CXR) were excluded. The rate of discontinuation of antibiotics, excluding azithromycin and doxycycline, in patients with a positive RVP with and without a bacterial co-infection were analyzed.

Results

Of these 92 patients, we found that a bacterial co-infection was detected by sputum culture in 20 patients. The average number of days until discontinuation for patients with no bacterial coinfection was 1.67 days while for those with a bacterial co-infection was 3.20 days. The difference in the number of days was statistically significant (p<0.001).

Conclusion

In conclusion, patients with an identified viral etiology of COPD exacerbations had antibiotics discontinued significantly sooner than those patients with bacterial coinfections.

## Introduction

Viral infections cause about 60% of chronic obstructive pulmonary disorder (COPD) exacerbations while bacterial infections account for about 40% of COPD exacerbations [1]. The viral causes of COPD exacerbations seldom require antibiotics but specific viral etiologies, such as influenza, require oseltamivir. Patients admitted for COPD exacerbations often are treated with antibiotics for presumed pneumonia or possibly for their anti-inflammatory effects. These events contribute to increased health care costs and the progressive deterioration of a patient’s health care status [1]. This study is aimed at observing the utilization of the respiratory viral polymerase chain reaction (RVP) and the subsequent discontinuation of antibiotics in patients who have COPD exacerbations caused by viral infections. Patients presenting with acute exacerbation of COPD (AECOPD), who had a positive RVP indicating a viral cause of their COPD exacerbation, were analyzed in this study to assess the use of antibiotics in our institution.

The identification of a viral pathogen for a patient’s COPD exacerbation could lead to a reduction in cost and length of stay, limit adverse medication effects, and mitigate other workups along with the discontinuation of antibiotics and prompt supportive care. The reduction in antibiotic use and the appropriate supportive care for viral COPD exacerbations should be an ongoing effort with this type of analysis. With the use of the RVP, we can identify viral pathogens that can promote antibiotic stewardship and appropriate isolation precautions. The aim of this study was to observe the use of antibiotics and the rate of discontinuation of antibiotics in patients with a viral cause of their COPD exacerbation.

## Materials and methods

In this observational study, we conducted a retrospective chart review of patients with an acute exacerbation of COPD and antibiotic use. We included patients admitted to St. Mary’s Hospital from July 1, 2017, to April 20, 2018. Inclusion criteria were adult male and female patients aged 18 to 85 years with a diagnosis of COPD exacerbation and a positive RVP. Exclusion criteria were a positive infiltrate seen on a chest radiograph. A total of 92 patients were included in this analysis, of which 20 patients had a bacterial co-infection based on sputum culture, which was collected on the day of admission. Sputum gram stains were available within one day of admission, and cultures were available within two days of admission. Azithromycin and doxycycline, typically given for the adjunctive treatment of COPD exacerbations, were not included in the antibiotic discontinuation analysis. In our study, 59/92 (64%) received either azithromycin or doxycycline for the purpose of their anti-inflammatory effects. This study was approved by the Trinity Health of New England Institutional Review Board.

Data collection

The following parameters were collected on chart review. The patient’s age, positive history of COPD, use of azithromycin or doxycycline in the acute setting, the use of other antibiotics, number of days until the discontinuation of antibiotics, a positive result of the RVP, date of RVP, sputum culture, and absence of a chest X-ray infiltrate.

Statistical analysis

Descriptive statistics included frequencies with percentages for qualitative variables and means with standard deviations for quantitative variables. To compare groups on the number of days until discontinuation, a Poisson regression model for count data was used. Analyses were conducted in SPSS v25 (IBM Corp., Armonk, NY) and the level for statistical significance was set at 0.05.

## Results

A total of 92 patient charts were included in the study, and 20 patients had a bacterial co-infection. In 28 out of 72 patients (38.9%), antibiotics were discontinued on the same day. Within the first two days, 47 out of 72 patients (65.3%) had antibiotics discontinued. By day eight, 100% of the patients were off antibiotics (Table 1).

**Table 1:** The frequencies of the number of days until the discontinuation of antibiotics in patients with a respiratory viral panel without a bacterial co-infection

Days	Frequency	Percentage	Cumulative Percent
0	28	38.9	38.9
1	19	26.4	65.3
2	8	11.1	76.4
3	3	4.2	80.6
4	6	8.3	88.9
5	2	2.8	91.7
6	2	2.8	97.2
7	2	2.8	97.2
8	2	2.8	100

**Figure 1:**
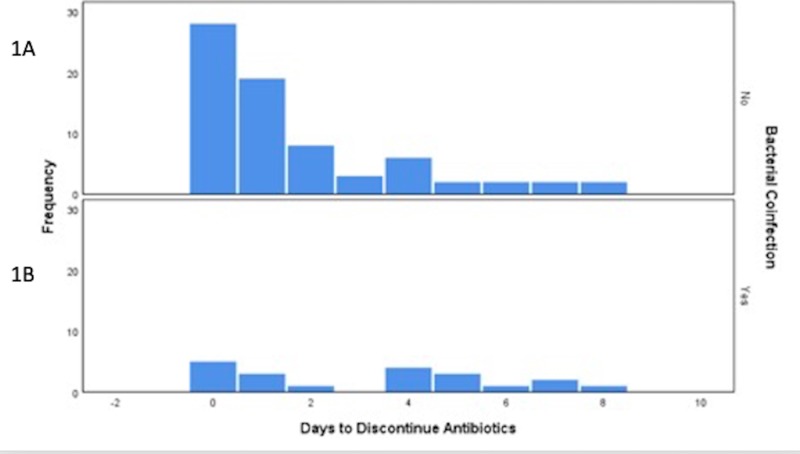
Distribution of number of days to discontinuation of antibiotics by whether patients had a bacterial coinfection 1A represents the days to the discontinuation of antibiotics in patients without a bacterial coinfection. 1B represents the days to the discontinuation of antibiotics with a bacterial coinfection.

The distribution of the number of days until discontinuation by bacterial coinfection status is reflected in Figure 1. The mean number of days to the discontinuation of antibiotics in patients with a viral infection causing a COPD exacerbation was 1.67 days (SD = 2.13) while for those with a bacterial coinfection, it was 3.20 days (SD = 2.71). The difference in the number of days was statistically significant (p<0.001). There were more patients without a coinfection, and those with a coinfection were less likely to be discontinued in 0 or 1 day.

We further stratified infections by month and found that in the months of September, October, and November, the average number of days to antibiotic discontinuation was the highest (Table 2). The fall season (September to November) had a longer time until discontinuation (Mean=3.21, SD=2.81) as compared to winter (Mean=1.29, SD=1.49), spring (Mean=1.26, SD=1.99), and summer (Mean=1.67, SD=1.16).

**Table 2:** The average (mean) number of days to discontinuation by month in patients with a positive respiratory viral panel without a bacterial co-infection

Month	Patients	Mean	SD
January	3	0.67	0.58
February	5	1.40	1.67
March	16	1.63	2.16
April	15	1.13	1.96
May	3	0.00	0.00
June	0		
July	0		
August	3	1.67	1.16
September	1	4.00	
October	4	3.50	3.00
November	9	3.00	3.04
December	13	1.38	1.61

The most common virus encountered was all the influenza subtypes and, incidentally, this was associated with the least number of days on antibiotics (Table 3). Nine patients (45%) had antibiotics discontinued on day zero, which is the highest percentage. There was a significant difference in the number of days until discontinuation by virus type (p=0.003).

**Table 3:** The average (mean) number of days to discontinuation by virus type

Virus	Patients	Mean	SD	Minimum	Maximum
Human Metapneumovirus (HMPV)	8	0.75	1.39	0	4
Influenza	20	1.45	1.96	0	7
Parainfluenza	8	3.25	2.76	0	8
Rhinovirus	19	1.95	2.55	0	8
Respiratory Syncytial Virus (RSV)	13	1.46	1.45	0	5
Multiple Viruses	4	0.75	1.50	0	3

## Discussion

AECOPD is a common cause of hospital admissions, which inflicts a substantial burden of morbidity, mortality, and health care costs [2]. The prevalence of respiratory viruses detected in patients with COPD vary. Hurst et al. report that respiratory viruses account for around 30% of exacerbations, whereas Clark et al. report that respiratory viruses are detected in 22%-44% in patients with COPD exacerbations [3]. In another study by Dimpolous et al., viral infections were associated with up to 60% of COPD exacerbations [1].

Antibiotic resistance is a worldwide public health issue that requires antibiotic stewardship, international attention, and ongoing efforts in mitigating the effects of emerging resistance [4]. Rhode et al. report that bacterial pathogens are absent in about 50% of COPD exacerbations, highlighting the importance of recognizing viral and other non-infectious etiologies of COPD exacerbations [5].

The recognition of clinical characteristics and the use of the RVP providing important clinical data in patients with AECOPD is an important issue highlighted in this study. Antibiotics have been considered part of the treatment for patients with an acute exacerbation of severe COPD, which includes increased sputum purulence and worsening shortness of breath, however, this population has not been studied if a positive RVP is noted. The RVP in our institution detects the following viruses: Human metapneumovirus, respiratory syncytial virus (RSV) subtypes, influenza subtypes, rhinovirus, parainfluenza subtypes, and adenovirus.

We observed patients with viral and bacterial coinfections as a cause of AECOPD and the use of antibiotic therapy from July 1, 2017, to April 20, 2018. We decided to exclude the use of azithromycin or doxycycline given ongoing studies of these antibiotics and their role in managing severe COPD exacerbation in the acute or chronic setting through their anti-inflammatory effects [4].

In patients with AECOPD, a positive RVP, and bacterial coinfection, the mean number of days to the discontinuation of antibiotics was 3.20 days. In patients without a bacterial co-infection, the mean days to the discontinuation of antibiotics was 1.67 days. The duration of antibiotics was found to be significantly less in the viral group alone, with a statistically significant difference in the number of days to discontinuation (p<0.001). Possible reasons for why antibiotics were discontinued on days zero to two in patients noted to have a bacterial co-infection may have been due to the patient’s clinical improvement or the patients were suspected to have colonizing bacteria based on their clinical history.

In the months of September through November, patients with a viral infection alone were treated with antibiotics for a longer duration as compared to the other months. We noticed that patients with influenza had the highest percentage of antibiotics discontinued on the first day of hospitalization but some remained on antibiotics for up to a week. It is known that viral infections are more prevalent in winter months and are associated with longer periods of recovery, as cold weather can cause a reduction in lung function, which may increase vulnerability to pollutants and viruses to further increase the risk of an AECOPD [1-2].

**Figure 2:**
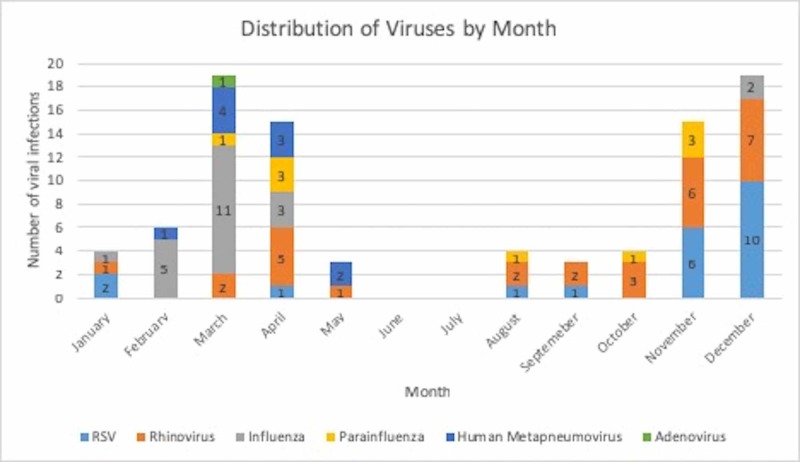
Distribution of viruses by month

Patients with parainfluenza had the highest average with a mean of 3.25 days before antibiotic discontinuation, followed by rhinovirus with a mean of 1.95 days before antibiotic discontinuation. Rhinovirus is one of the more commonly identified viruses in AECOPD and a known etiology of the common cold [2,6]. This was consistent with our study, where rhinovirus was noted to be the most prevalent in our patient population throughout the year, with a noticeable increase in the winter months. A total of 29 (31.5%) cases of rhinovirus were detected in our patient population (Figure 2).

Early testing for viral infections is optimal for ensuring appropriate management [7]. In our study, the RVP was obtained within 24 hours of admission. The limitation of the RVP at our institution is that it takes two to three hours to get a result, therefore, it is not a preferred test in the emergency department. In addition, the RVP is not processed during the night shift hours of 7 pm to 7 am. These limitations of the RVP, as well as the available results of sputum cultures, influence the decision of how quickly to discontinue antibiotics. The challenge of coinfections with bacteria is whether patients with COPD are colonized with bacteria in their respiratory tract or have a true bacterial coinfection.

Our study is not without limitations, as this was a retrospective single-center study. Exacerbations of COPD were not stratified by severity. Some patients who were continued on antibiotics may have had other indicators suggestive of a bacterial pulmonary infection, such as elevated white blood cell count or fever, which prompted the continuation of antibiotic therapy [7]. We did not account for the severity of infection in these patients regarding the need for mechanical ventilation or a critical level of care. Our study population was limited to one center with a small population, which limits generalizability and applicability to large populations. Our study does not address the sensitivity or specificity of the RVP, nor does it address clinical outcomes if antibiotics were discontinued sooner with the use of the RVP. These observed limitations offer potential avenues for further study.

## Conclusions

In conclusion, the time to the discontinuation of antibiotic therapy in patients with AECOPD with an isolated viral etiology was 1.67 days, while for those with a bacterial co-infection, it was 3.20 days. The difference in the number of days was statistically significant (p<0.001). Though 20 (21.7%) patients had bacterial coinfections, the majority of patients did not require antibiotics because of the detection of a single viral etiology. Therefore, the identification of a viral etiology can avoid unnecessary antibiotic usage thereby minimizing antibiotic resistance. The use of the respiratory viral panel may encourage antibiotic stewardship in this patient population. However future studies would be needed to ascertain data regarding antibiotic stewardship. Given the findings of this study, we encourage stopping antibiotics sooner if the RVP is positive, sputum cultures are negative, and a chest X-ray is normal. Further studies are needed to observe the rate of discontinuation of antibiotics after the identification of a viral etiology and its impact on clinical outcomes, including the length of hospital stay as well as the risk of readmission. In addition, future studies could analyze these two subsets of patients after the withdrawal of antibiotics and the impact of clinical deterioration requiring a higher level of care, need for intubation or noninvasive positive-pressure ventilation (NIPPV), adverse drug effects, death and re-initiation of antibiotics in multiple centers to improve the applicability of antibiotic stewardship in this subset of patients.
